# A genome scan for quantitative trait loci affecting cyanogenic potential of cassava root in an outbred population

**DOI:** 10.1186/1471-2164-12-266

**Published:** 2011-05-25

**Authors:** Sukhuman Whankaew, Supannee Poopear, Supanath Kanjanawattanawong, Sithichoke Tangphatsornruang, Opas Boonseng, David A Lightfoot, Kanokporn Triwitayakorn

**Affiliations:** 1 Institute of Molecular Biosciences, Mahidol University, Nakhon Pathom, Thailand; 2 National Center for Genetic Engineering and Biotechnology, Bangkok, Thailand; 3 Center for Cassava Molecular Biotechnology, Faculty of Science, Mahidol University, Bangkok, Thailand; 4 Rayong Filed Crops Research Center, Ministry of Agriculture and Cooperatives, Rayong, Thailand; 5 Plant Biotechnology and Genomics Core-Facility, Department of Plant, Soil, and Agricultural Systems, Southern Illinois University, Carbondale, IL 62901, USA

## Abstract

**Background:**

Cassava (*Manihot esculenta *Crantz) can produce cyanide, a toxic compound, without self-injury. That ability was called the cyanogenic potential (CN). This project aimed to identify quantitative trait loci (QTL) associated with the CN in an outbred population derived from 'Hanatee' × 'Huay Bong 60', two contrasting cultivars. CN was evaluated in 2008 and in 2009 at Rayong province, and in 2009 at Lop Buri province, Thailand. CN was measured using a picrate paper kit. QTL analysis affecting CN was performed with 303 SSR markers.

**Results:**

The phenotypic values showed continuous variation with transgressive segregation events with more (115 ppm) and less CN (15 ppm) than either parent ('Hanatee' had 33 ppm and 'Huay Bong 60' had 95 ppm). The linkage map consisted of 303 SSR markers, on 27 linkage groups with a map that encompassed 1,328 cM. The average marker interval was 5.8 cM. Five QTL underlying CN were detected. *CN08R1*from 2008 at Rayong, *CN09R1*and *CN09R2 *from 2009 at Rayong, and *CN09L1 *and *CN09L2 *from 2009 at Lop Buri were mapped on linkage group 2, 5, 10 and 11, respectively. Among all the identified QTL, *CN09R1 *was the most significantly associated with the CN trait with LOD score 5.75 and explained the greatest percentage of phenotypic variation (%Expl.) of 26%.

**Conclusions:**

Five new QTL affecting CN were successfully identified from 4 linkage groups. Discovery of these QTL can provide useful markers to assist in cassava breeding and studying genes affecting the trait.

## Background

Throughout the world, cassava (*Manihot esculenta *Crantz) has been cultivated as an important food source and industrial feedstock since agriculture was developed. Accordingly, it ranked fourth among all crops in worldwide production. Thailand was the world's leading exporter [1]. As a cyanogenic crop, cassava has the ability to release hydrogen cyanide (HCN) during cell damage [2,3]. Variation among cultivars in their cyanogenic potential (CN), causes concerns about their possible health effects such as acute intoxication, manifested as vomiting, dizziness, etc. [4] and environmental toxicity [5]. Improved cassava cultivars with low CN and an improved understanding of genes affecting CN were of considerable interest.

CN was reported to be a quantitative trait [5]. Since conventional breeding was not effective or efficient with quantitative trait loci (QTL) of moderate to low heritability, molecular breeding was expected to be an efficient, reliable and cost effective breeding approach [6,7]. For molecular breeding, QTL analysis was often used to identify trait-linked markers in order to facilitate marker-assisted selection (MAS). QTL identification was not only used to assist breeding program, but also to gain understanding of the loci and underlying genes and their effects [6,7].

In the present study, an F_1 _outbred population was chosen for QTL analysis because that population structure required the least time to generate. The population provided the significant segregation of both genotype and phenotype as required for QTL discovery [8-10]. A large number of SSR markers for cassava have been developed [11-14]. These markers were used to construct a genetic linkage map in order to apply for identification of QTL underlying CN trait.

## Results and Discussion

### Phenotypic measurement

Variation in the CN in an outbred population derived from 'Hanatee' × 'Huay Bong 60' and its parents are shown in Table 1. In all evaluated years and locations, the CN of 'Hanatee' was approximately two folds lower than 'Huay Bong 60'.

**Table 1:** Phenotypic values of outbred population and their parents.

		Cyanogenic potential (ppm)*			
		
		2008 at Rayong	2009 at Rayong	2009 at Lop Buri	Mean
	Hanatee	40.8 ± 11.0	22.8 ± 11.0	35.7 ± 16.6	33.3
Mean	Huay Bong 60	106.1 ± 40.7	57.9 ± 32.9	131.4 ± 39.7	95.0
	F1 progenies	80.3 ± 24.4	35.0 ± 15.5	48.9 ± 21.5	55.2

Maximum		146.9	83.9	104.2	115

Minimum		38.4	10.8	14.3	25

Mean values of cyanogenic potential plus or minius SEMs in the parents ('Hanatee' and 'Huay Bong 60') and their progenies from 2008 at Rayong, 2009 at Rayong and 2009 at Lop Buri, and maximum and minimum trait values of the progeny population

* mg HCN equivalents kg^-1 ^fresh weight (ppm)

The distribution of the CN in the population showed continuous variation, across a wide range (Table 1), typical of quantitative traits. That implied that CN would be underlain by polygenes. Transgressive variation was observed that may have resulted from cooperation or interaction among the loci and genes present in the two parental types. In addition, over-dominance and epitasis may also have contributed to the transgressive segregation [15]. The correlation coefficients (r) among years and locations ranked from 0.308 to 0.487 and showed significance at *P *< 0.01 (Table 2). Therefore, the phenotypic data were appropriate for QTL analysis but mean data might be less informative than individual environments.

**Table 2:** The correlation coefficients

	2008, Rayong	2009, Rayong	2009, Lop Buri
2008, Rayong	1		
2009, Rayong	0.413 (**)	1	
2009, Lop Buri	0.308 (**)	0.487 (**)	1

The correlation coefficients of cyanogenic potential among the lines of the population derived from 'Hanatee' × 'Huay Bong 60' in 2008 at Rayong, in 2009 at Rayong and in 2009 at Lop Buri

** = Significant at *P *< 0.01

### Linkage map construction

A total of 1,732 available SSRs consisting of 667 primer pairs provided from the International Center for Tropical Agriculture (CIAT) [16], 425 primer pairs from 640 primer pairs from Sraphet et al. (2011) [12] and Kunkeaw et al. (2011) [14] were tested for informative markers between the parental lines. Of these, 507 markers (28.9%) were informative and successfully genotyped within the population. From genotypic data using 507 loci, the results showed that 151 markers (~30%) had distorted segregation ratios. Eleven markers (~2%) were 100% identical to other marker loci. Therefore, those 162 total DNA markers were excluded. The highly distorted segregation ratios found in this study were common among out-crossing species like cassava [17]

**Figure 1:**
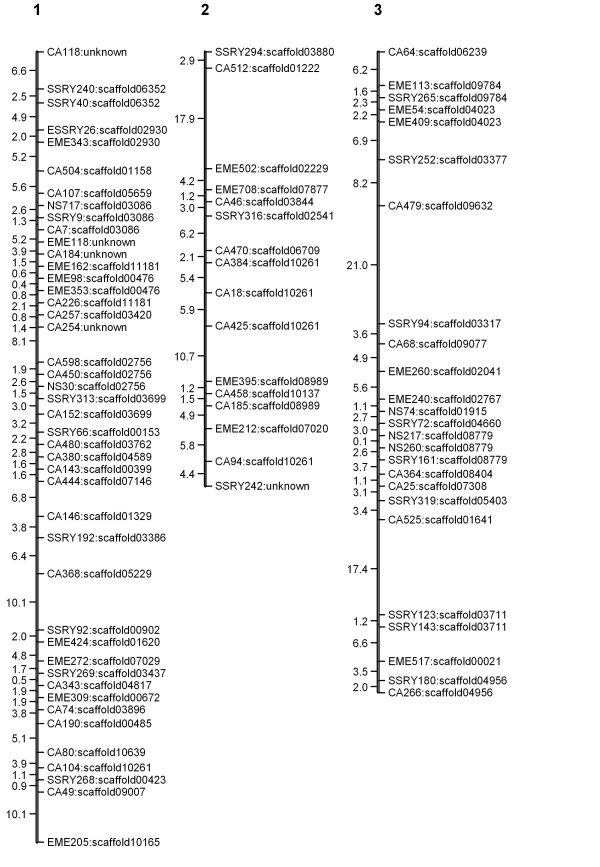
**Genetic linkage map of cassava based on SSR marker (LG1-3)**. The numbers above each bar indicated linkage group name. To the left hand side of each bar the number indicated interval distance in cM. To the right hand side of each bar the number indicated locus name and scaffold of cassava genome that was anchored.

**Figure 2:**
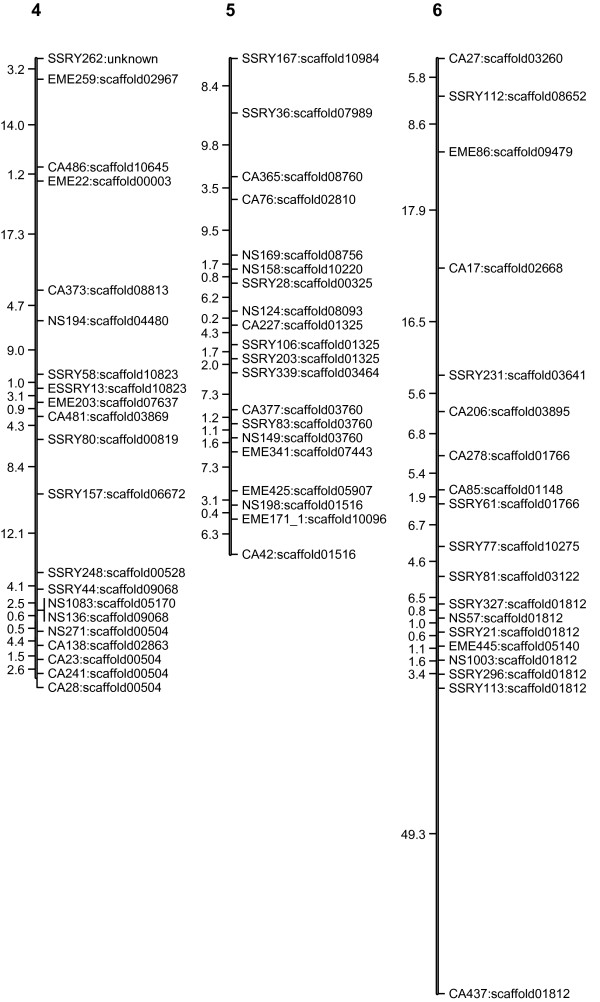
**Genetic linkage map of cassava based on SSR marker (LG4-6)**. The numbers above each bar indicated linkage group name. To the left hand side of each bar the number indicated interval distance in cM. To the right hand side of each bar the number indicated locus name and scaffold of cassava genome that was anchored.

**Figure 3:**
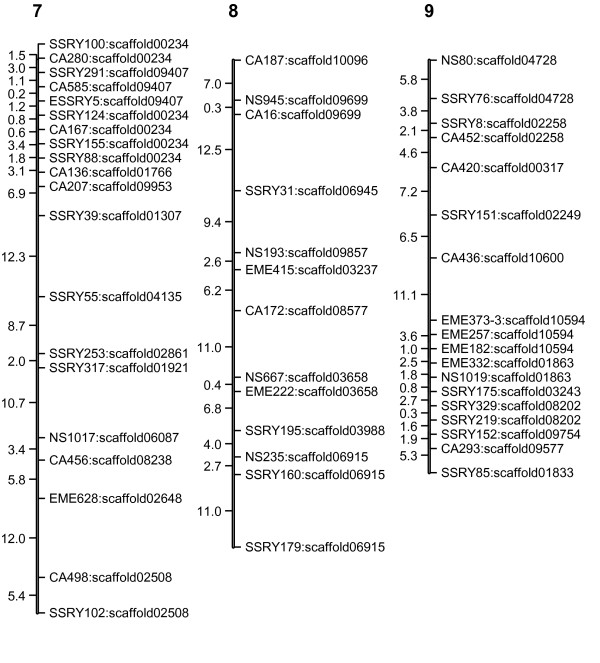
**Genetic linkage map of cassava based on SSR marker (LG7-9)**. The numbers above each bar indicated linkage group name. To the left hand side of each bar the number indicated interval distance in cM. To the right hand side of each bar the number indicated locus name and scaffold of cassava genome that was anchored.

**Figure 4:**
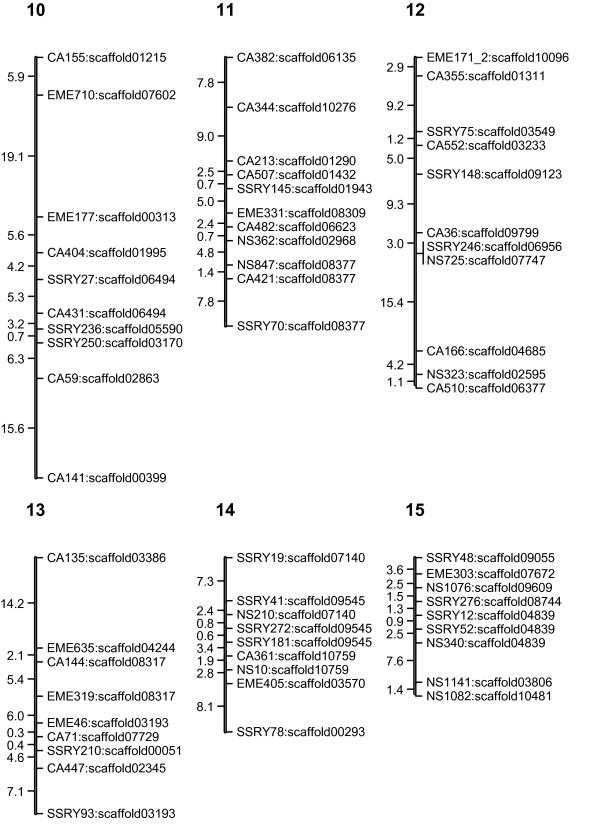
**Genetic linkage map of cassava based on SSR marker (LG10-15)**. The numbers above each bar indicated linkage group name. To the left hand side of each bar the number indicated interval distance in cM. To the right hand side of each bar the number indicated locus name and scaffold of cassava genome that was anchored.

**Figure 5:**
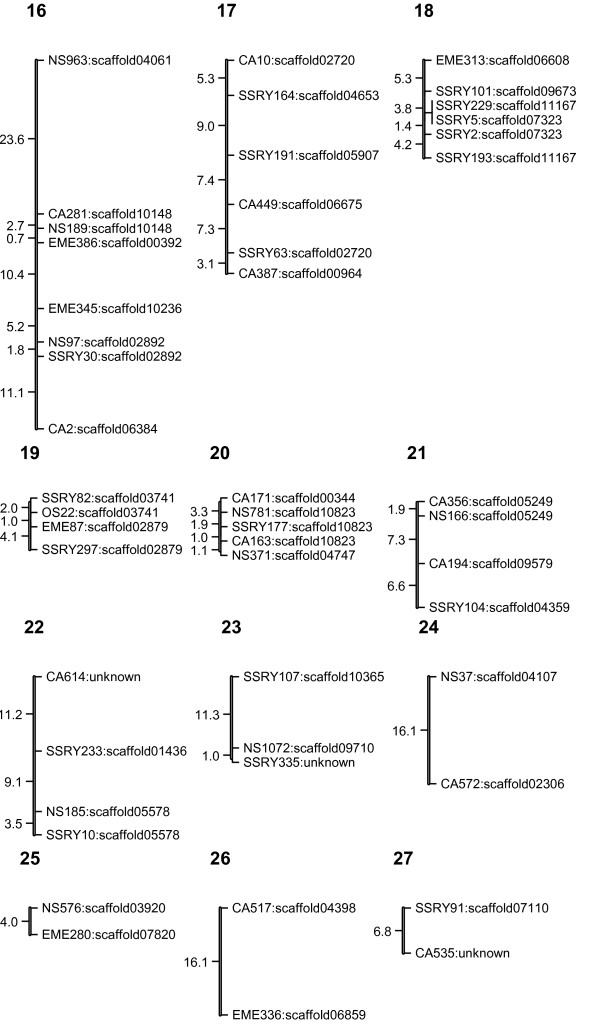
**Genetic linkage map of cassava based on SSR marker (LG16-27)**. The numbers above each bar indicated linkage group name. To the left hand side of each bar the number indicated interval distance in cM. To the right hand side of each bar the number indicated locus name and scaffold of cassava genome that was anchored.

The linkage map (Figures 1, 2, 3, 4 and 5) consisted of 303 markers located on 27 linkage groups. The map covered 1,328 cM with average spacing between markers of 5.8 cM, smaller than the 10 cM desirable for QTL detection by interval mapping (IM) [18-20]. Although the derived map had good potential to identify QTL, it was not yet a saturated map. More markers or inclusion of markers with distorted segregation ratios maybe required, to bring the number of linkage groups equal to the number of haploid genome (n = 18).

The cassava genome database contained a draft sequence in late 2010 [21], in which there were 11,243 scaffolds spanning 416 Mb. The SSR loci used in the map were identified in the scaffolds. A total of 220 scaffolds were placed on the map. Of these, 50 scaffolds containing more than one marker locus from the same linkage group. However, 13 of 50 scaffolds were separated by markers from other scaffolds. For example, on the LG1, there were 44 loci which can be located on 31 scaffolds of the cassava genome sequences (Figures 1, 2, 3, 4 and 5). Within the region between EME162 to CA226, scaffold11181 was separated by scaffold00476. The lack of correspondence of some marker orders in our genetic map with the physical order in the sequence of cassava may have resulted from inaccurate estimations of genetic distance based on recombination frequency. Errors might be found in both genetic maps and preliminary scaffold assemblies. Alternately, the difference in genetic background of the cassava used in the two studies may underlie the differences.

### QTL discovery

Based on multiple-QTL models (MQM) analysis, only 5 QTL were detected across 4 linkage groups, 1-2 from each environment (Table 3). Each QTL showed LOD scores that were higher than the chromosome wide significant threshold. The LOD scores of the QTL identified ranged from 3.77-5.75. The QTL with the largest effect had a LOD score higher than both chromosome and genome wide significant thresholds. *CN09R1 *was located on linkage group 10 associated with marker locus CA141. In addition, *CN09R1 *also explained the largest portion of variation at 26.

**Table 3:** Description of five QTL for cyanogenic potential in cassava and their functional annotation

Environments	ag	QTLs	Loci	LG	ac	LOD	%expl	Phenotype mean (ppm)*				Location on physical map	Functional annotation
								
								ac	ad	bc	bd		
Rayong, 2008	4.4	*CN08R1*	CA227	5	3.0	3.77	16.1	92.0	72.2	70.4	88.3	Scaffold08093 at 10.8 kbp	Unknown

Rayong, 2009	4.5	*CN09R1*	CA141	10	2.8	5.75	26.0	23.4	29.6	26.5	45.6	Scaffold00399 at 564.7 kbp	Unknown
		
		*CN09R2*	CA344	11	2.4	4.05	15.9	23.4	31.2	20.1	36.5	Scaffold10276 at 15 kbp	Nucleotide-binding protein of 35 kDa

Lop Buri, 2009	4.4	*CN09L1*	CA18	2	3.0	4.58	23.0	62.3	50.4	80.3	61.9	Scaffold10261 at 564 kbp	β-1,3-N-Acetylglucosaminyl transferase
		
		*CN09L2*	CA76	5	3.2	4.22	18.5	62.3	38.2	41.6	51.9	Scaffold02810 at 207.3 kbp	Adenosine/Guanosine diphosphatase

Genetic metrics for each QTL: Detail of each QTL affecting cyanogenic potential from evaluating in 2008 at Rayong (RY08), 2009 at Rayong (RY09) and 2009 at Lop Buri (LB09)

αc Chromosome wide significance threshold

αg Genome wide significance threshold

%expl Percentage of phenotypic variation explain

* mg HCN equivalents kg^-1 ^fresh weight (ppm)

The LOD scores of some QTL were lower than genome wide significant threshold as expected for sparse maps (maps with marker density > 1 cM) [22,23]. However, for all QTL, the phenotype means indicated that one allele combination was markedly better at each location although the differences were not all significant. Therefore, many QTL were dependent on the environment. Equally, the large variation in CN found among environments showed a strong environmental effect (Table 2). The percentage of phenotypic variation explained (%Expl.) from all detected QTL ranged from 15.9-26.0% (Table 3).

**Figure 6:**
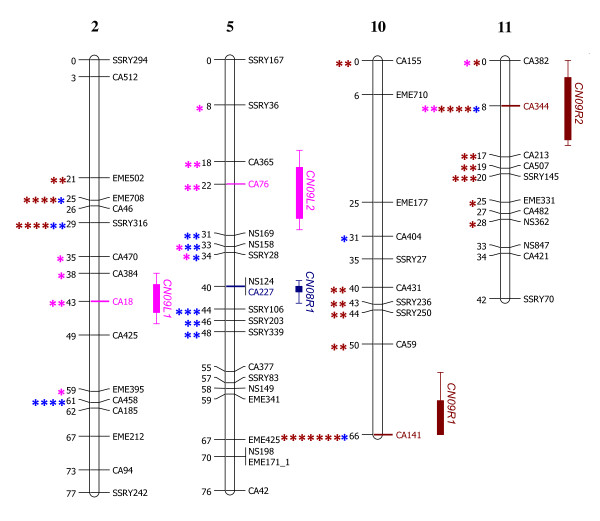
**Locations of 5 QTL underlying cyanogenic potential**. Box-bars with extending lines at the right side of the linkage group indicated the region within 1and 2 LOD units of the peak. The QTL names are on the right of each box-bar. The significance of Kruskal-Wallis analyses are shown at the left side of the linkage group (* is *P *≤ 0.1, ** is *P *≤ 0.05, *** is *P *≤ 0.01, **** is *P *≤ 0.005, ******* is *P *≤ 0.00001). All symbols colored with blue, red and pink, refer to the analysis in 2008 at Rayong, 2009 at Rayong and at Lop Buri, respectively.

Although MQM showed the highest precision to identify and map QTL, Kruskal-Willis (KW) analysis was used for single marker analysis to avoid type 2 errors. Except for *CN08R1*, all identified QTL showed significance by KW analyses (Figure 6). Even though *CN08R1 *did not show significance by KW analysis, loci flanking this QTL showed strong significance. That suggested that all loci detected were the real QTL not type 1 errors [6,24]. No common QTL was found across all environments (Table 3), which may be because the CN was highly sensitive to environment [4]. However, KW analysis of *CN09R2 *showed significant association with CN across all environments.

In a previous study by Kizito et al. (2007) [25] two QTL for CN were found linked to loci SSRY105 and SSRY242. The locus SSRY105 was not polymorphic in the current map, but SSRY242 was found on linkage group 2 as was *CN09L1*. However, SSRY242 was 34.4 cM far from the position of *CN09L1*. It should be kept in mind that QTL analysis is based on statistical analysis and there is a strong environmental effect on CN [4]. Therefore, individual QTL may not be found only in specific populations or environments. However, this study helped develop markers to assist in cassava improvement, and for the study of genes affecting to CN and for comparative mapping in other related species.

In addition, functional gene annotation of the regions within 2-LOD support of each QTL was performed. A total of 159 annotated genes, consisting of 4, 18, 19, 106 and 12 predicted genes underlying the regions of *CN08R1, CN09R1, CN09R2*, *CN09L1 *and *CN09L2*, respectively were identified (Additional file 1). Interestingly, three annotated genes at the QTL peaks have been predicted including; a nucleotide-binding protein of 35 kDa (Nbp35; EC 3.6.1.15), β-1,3-N-acetylglucosaminyl transferase (EC 2.4.1.149) and an adenosine/guanosine diphosphatase (EC 3.6.1.6) within intervals that contained QTL *CN09R2*, *CN09L1 *and *CN09L2*, respectively (Table 3). Nbp35 belongs to subgroup of the P loop NTPases which perform a wide variety of cellular functions such as signal transduction, signal-sequence recognition, protein transport and localization, chromosome partitioning, etc. [26]. Nbp35 was an iron-sulfur protein with a dual localization in the cytosol and nucleus. It played a direct role in biogenesis and was essential for eukaryotic metal ion insertion in proteins and membrane transport [27].

Adenosine/guanosine diphosphatases were nucleoside diphosphatase acting on hydrolysis of phosphoric ester. One of the substrates for this enzyme was nucleoside diphosphate which is generated by glycosyltransferase in the fungal, plant, and mammalian cell secretory pathways [28]. The conversion of nucleoside diphosphate by nucleoside diphosphatase yields monophosphates which relieve inhibition of the transfer enzymes and provide substrates for antiport transport systems by which the entrance of nucleotide sugars from the cytosol into the secretory pathway lumen was mediated. However, the absence of diphosphatase activity does not end glycosylation or the entrance of nucleotide sugars into lumen [29].

β-1,3-N-Acetylglucosaminyl transferase belongs to the glycosyltransferase family of enzymes. They act to catalyze the transfer of a sugar (monosaccharide) unit from a sugar nucleotide derivative to a sugar or amino acid acceptor (EC2.4.-) [30]. Cyanogenic glycosides are precursors of HCN in cyanogenesis pathway [31]. To produce linamarin and lotaustralin, which are cyanogenic glycosides in cassava, the glcosyltransferase must catalyze L-valine and L-isoleucine [32], and thus the gene encoding this β-1,3-N-acetylglucosaminyl transferase might be involved in this process.

There is no report to link Nbp35 and Adenosine/Guanosine diphosphatase directly to the cyanogenesis pathway, however these two enzymes were involved in common biogenesis. In addition, sequences of linamarase (EC 3.2.1.21) and hydroxynitrile lyase (4.1.2.11) which are key enzymes involved in the cyanogenesis pathway were found on scaffold09743 and scaffold01206, respectively. However, these scaffolds were not anchored by any of the markers in this study linkage map. Thus, it would be useful to identify additional markers to link these enzymes to the map.

## Conclusions

In this study, an SSR based genetic linkage map of cassava was constructed using an F_1 _population of a cross between 'Hanatee' and 'Huay Bong 60'. The map was used for analysis of QTL underlying CN, and five potential QTL were detected. Among all the QTL, *CN09R1*, which was located on linkage group 10 was the strongest with the LOD value of 5.75 and it explained 26.0% of the variation in CN. In addition, all loci on the genetic map were compared with the data from cassava genome sequence. The anchor markers common to both could help the organizing and completion of chromosome sized scaffolds. The loci found in this study will be useful for identification of genes controlling the traits as well as establishing MAS of cassava in the future.

## Methods

### Plant materials and field experiment

Cassava variety 'Hanatee' (Thai local variety), exhibited low CN and 'Huay Bong 60' (commercial variety) [33], displayed high CN. They were used as female and male parent, respectively. A hundred of their progeny were used for the mapping population.

All samples were planted in May during years 2007-2008. In 2007, the population and its parents were separately cultivated at Rayong province, Thailand. Ten cutting stems per genotype were planted with ten rows at a space of 1 × 1.5 m. In 2008, they were cultivated at Rayong and Lop Buri provinces, Thailand. Each genotype was replicated two times in 10 × 10 simple lattice designs at a space of 0.8 × 1 m. Fertilizer (N:P:K; 15:15:15), 312.5 kg/Hectare and chicken manure, 3,100 kg/Hectare were applied at one month after planting. Pest management was applied as necessary. Roots were harvested for CN evaluation at one year after planting. The climate at both locations was warm and humid all year round with an average temperature of 28°C. The average rainfall at Rayong and Lop Buri was 1,339.4 and 1,211.9 mm per year [34], respectively. There were different soil types at Rayong (clayey loam soil) and Lop Buri (clay soil) [35]. There was no evidence of pests and diseases that occurred in the planted areas.

### Monitoring the CN

The CN was evaluated in roots as mg HCN equivalents kg^-1 ^fresh weight (ppm) using picrate paper kit as described by Bradbury et al. (1999) [36]. The roots of the population and its parents were harvested in 2008 at Rayong, and in 2009 at Rayong and at Lop Buri. In 2008 at Rayong, three plants of each genotype were selected, and three roots of each plant were collected. In 2009 at Rayong and Lop Buri, two plants of each genotype of each replication were used.

### SSR analysis

Genomic DNA of the population and its parents was extracted from young leaves based on CTAB selective precipitation of DNA, modified according to Fulton et al. (1995) [37]. A total of 1,732 SSRs were used consisting of 667 SSRs provided by CIAT, 640 SSRs from Sraphet et al. (2011) [12] and 425 SSRs from Kunkeaw et al. (2011) [14]. They were screened against the parents to find informative markers used to genotyped the F_1 _population. The PCR reactions were carried out in 20 μl final volume containing 50 ng of genomic DNA, 1 × PCR buffer (Promega, Madison WI, USA) with 1.5 mM MgCl_2_, 0.2 μM of each PCR primer, 200 mM of each dNTP and one unit of DNA-polymerase (Promega). The PCR program for SSR amplification consisted of the following steps: 94°C for 2 min followed by 35 cycles of 94°C for 30 s, 55°C for 45 s and 72°C for 1 min, then a final step of 72°C for 5 min (modified from Tangphatsornruang et al. (2008) [13]). Products were analyzed using 5% denaturing polyacrylamide gel electrophoresis and were visualized by silver staining according to Benbouza et al. (2006) [38]. The amplicon band patterns were scored according CP codes (eg. <abxcd>, <efxeg>, <lmxll>, <nnxnp> and <hkxhk>) and missing data was replaced by "--" as described by Ooijen and Voorrips (2001) [39].

### Linkage map construction

For linkage map construction, all genotypic data were loaded into JoinMap^® ^3.0 program software [39]. The program first tested the segregation ratio of each marker using chi-square (χ^2^) test. Statistically, significant markers at *P *< 0.05 were excluded from further analysis. The similarity of loci was then tested and markers showing 100% similarity were also excluded. The remaining markers were mapped into linkage groups based on the LOD threshold of 6.0 and maximum recombination threshold of 0.4. The genetic distance in a unit of recombinant frequency or centimorgan (cM) was calculated using Kosambi mapping function.

### QTL analysis

As a first step, the data file containing the marker observations, the mean trait values and the genetic linkage map (output from JoinMap^® ^3.0) were loaded into MapQTL^® ^4.0 program [9]. IM analysis was performed followed by permutation tests in order to determine the significance threshold of LOD score. The markers that showed LOD scores from IM higher than the chromosome wide threshold with *P *< 0.05 from the 1,000 permutations were selected as the cofactor. The automatic cofactor selection tool was used for selection of cofactors. They were selected by user before performed MQM mapping analysis. The markers that showed LOD score higher than the chromosome wide threshold with *P *< 0.05 from the 1,000 permutations were identified as QTL. KW analysis, a single marker analysis, was also analyzed based on one way ANOVA.

### Blast analysis and functional annotation

On-line BLAST against the cassava genome with primer sequences located on linkage map was performed on Phytozome [21]. Functional annotation of each QTL was identified based on the Panther classification system [30].

## Authors' contributions

SW and KT conceived of the study together with the other authors, carried out the major part of the experiments, analyzed the results and prepared the manuscript. SP and SK participated in the genotypic analysis. ST involved in the data analysis. OB prepared plant materials. DAL was involved in drafting the manuscript and revising it critically. All authors read and approved the final manuscript.

## Supplementary Material

Additional file 1 **List of annotated genes within 2-LOD support regions of the QTL**. Click here for file
